# Assessment of olfactory detection thresholds in children with autism spectrum disorders using a pulse ejection system

**DOI:** 10.1186/s13229-016-0071-2

**Published:** 2016-01-19

**Authors:** Hirokazu Kumazaki, Taro Muramatsu, Takashi X. Fujisawa, Masutomo Miyao, Eri Matsuura, Ken-ichi Okada, Hirotaka Kosaka, Akemi Tomoda, Masaru Mimura

**Affiliations:** Research Center for Child Mental Development, University of Fukui, 23-3, Matsuokashimoaizuki, Eiheiji-cho, Yoshida-gun, Fukui 910-1193 Japan; Department of Neuropsychiatry, Keio University School of Medicine, 35 Shinanomachi, Shinjuku-ku, Tokyo 160-8582 Japan; Donguri Psycho Developmental Clinic, Setagayaterasu # 1F, 4-14-5 Minamikarasuyama, Setagaya-ku, Tokyo 157-0062 Japan; Graduate School of Science and Technology, Keio University, 3-14-1 Hiyoshi, Kohoku-ku, Yokohama, Kanagawa 223-8522 Japan

**Keywords:** Autism spectrum disorder, Laboratory-based studies, Pulse ejection system, Olfaction, Olfactory detection threshold

## Abstract

**Background:**

Atypical responsiveness to olfactory stimuli has been reported as the strongest predictor of social impairment in children with autism spectrum disorders (ASD). However, previous laboratory-based sensory psychophysical studies that have aimed to investigate olfactory sensitivity in children with ASD have produced inconsistent results. The methodology of these studies is limited by several factors, and more sophisticated approaches are required to produce consistent results.

**Methods:**

We measured olfactory detection thresholds in children with ASD and typical development (TD) using a pulse ejection system—a newly developed methodology designed to resolve problems encountered in previous studies. The two odorants used as stimuli were isoamyl acetate and allyl caproate.

**Results:**

Forty-three participants took part in this study: 23 (6 females, 17 males) children with ASD and 20 with TD (6 females, 14 males). Olfactory detection thresholds of children with ASD were significantly higher than those of TD children with both isoamyl acetate (2.85 ± 0.28 vs 1.57 ± 0.15; *p* < 0.001) and allyl caproate ( 3.30 ± 0.23 vs 1.17 ± 0.08; *p* < 0.001).

**Conclusions:**

We found impaired olfactory detection thresholds in children with ASD. Our results contribute to a better understanding of the olfactory abnormalities that children with ASD experience. Considering the role and effect that odors play in our daily lives, insensitivity to some odorants might have a tremendous impact on children with ASD. Future studies of olfactory processing in ASD may reveal important links between brain function, clinically relevant behavior, and treatment.

## Background

A growing body of evidence suggests that children with autism spectrum disorders (ASD) experience increased sensory symptoms compared to children with typical development (TD) or those having general delays [1, 2]. The fact that recently released diagnostic criteria of the Diagnostic and Statistical Manual of Mental Disorders, 5th Edition (DSM-5) [3] have included sensory issues reflects the growing interest of these symptoms in ASD. Among sensory systems, an abnormal response to taste and smell has been reported to be the most pronounced when dissociating children with ASD from children with other developmental disorders [1, 4]. Moreover, differences in the olfactory traits of children with ASD might contribute to high rates of food refusal and selectivity [5, 6], and atypical responsiveness to olfactory stimuli have been reported as the strongest predictor of social impairment in children with ASD [7, 8].

Despite their importance, olfactory abnormalities in children with ASD are still poorly understood compared to abnormalities in touch, vision, and audition. In fact, there have been limited experimental studies concerning olfactory abnormalities in ASD. According to Dunn model [9], sensory modulation disorders (SMDs) are classified into three types: over-responsivity, under-responsivity, and sensory seeking. Among sensory symptoms, differences between individuals with ASD and TD subjects were greatest for under-responsivity, which describes a high threshold for sensory input [10, 11]. This trait is associated with lower adaptive functioning and poorer communication and social performance [8, 12]. Therefore, investigations of olfactory detection thresholds could provide important clues regarding the nature of children with ASD. However, previous laboratory-based sensory psychophysical studies [13–16] that have investigated olfactory detection thresholds in children with ASD have produced inconsistent results, probably due to methodological difficulties. The methods used in these studies were the University of Pennsylvania Smell Identification Test (UPSIT) [17], Sniffin’ Sticks [18], and the alcohol sniff test (AST) [19]. For these measurements, controlling scent granularity is a considerable challenge because of the problem of scent scattering in the air. The influence of lingering scent is a major problem [20]. Since olfaction is one of the most readily adaptable senses [21], accurate measurements are not possible when scents are left lingering in the air [22]. Concerning adaptation, the participants being exposed to the odor stimuli for a long time is also a serious problem inherent in these methods. To solve these problems, robust methodology is needed to measure thresholds of olfactory detection.

**Fig. 1:**
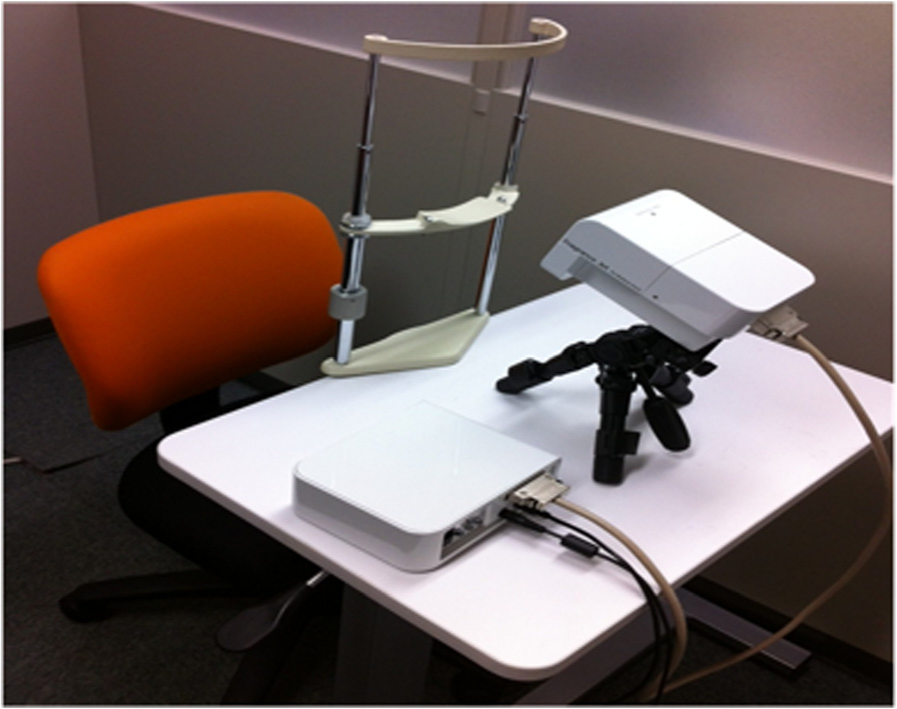
Fragrance jet for medical checkup. This device creates ejection pulses for scent presentation with a high degree of granularity control. In response to pulsed scents, the device can measure and quantify olfactory detection thresholds in precise detail

**Fig. 2:**
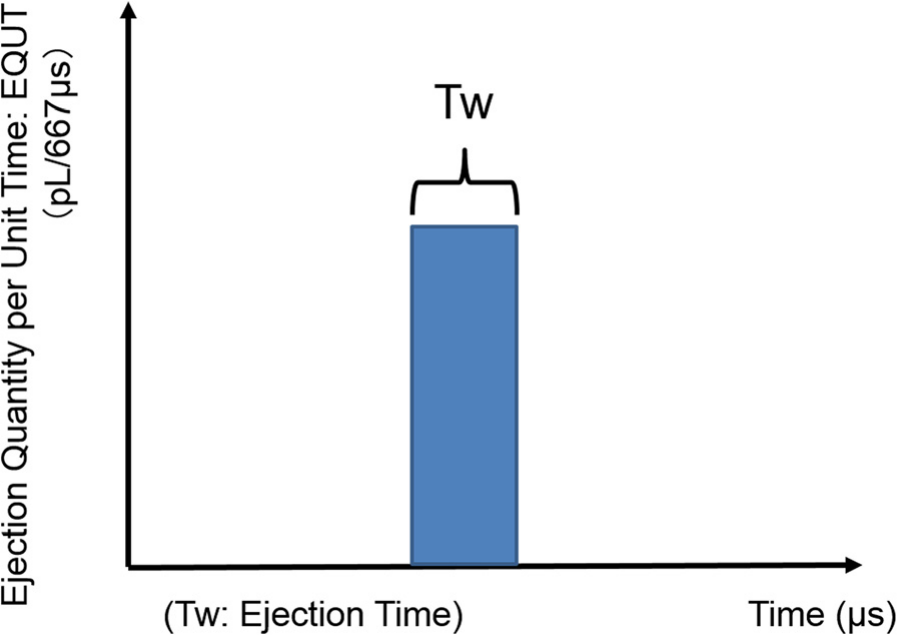
Conceptual graph of the pulse ejection system. The scent intensity is controlled by two parameters: ejection quantity per unit time (EQUT) and ejection time (ET). Ejection can be controlled in pulses of 667 μs. Compared to existing measurement techniques, this system can minimize lingering scent trails and makes it more difficult for the olfactory system to adapt to a particular odor

To address this issue, we developed a method called the Fragrance Jet for Medical Checkup (Keio University) (Fig. 1), which uses a pulse ejection system [20]. The system can measure and quantify the olfactory detection threshold in precise detail in response to pulsed scents. The test has been reliably standardized and is appropriate for both children and adults. It uses the same technique as used in a basic inkjet printer in that it outputs tiny droplets of fragrance. The system has one large tank and three small ones for olfactory measurement and produces a small jet that disperses into droplets from small holes in the tank. There are 255 minute holes in the large tank and 127 minute holes in small tanks. It is possible to emit scent at the same time through all these holes. Figure 2 shows a conceptual graph of a pulse ejection. The scent intensity is controlled by two parameters: ejection quantity (EQ) per unit time (EQUT) and ejection time (ET). The device can change the ET at 667-μs intervals so that the measurement can be precisely controlled. Unlike existing olfactory measurement techniques which change the concentration of a scent and require that these various concentrations be prepared ahead of time, our approach allows us to measure the olfactory detection threshold by only changing the EQ. Using very small quantities of an odorant reduces lingering scents during measurement and makes adaptation of the olfactory system more difficult [23]. Thus, along with using small odorant quantities, our measurements were performed in a location that was adequately ventilated in order to prevent lingering scent trails.

In addition to the pulse ejection system and experimental room conditions, several aspects of our system increase its efficiency and credibility relative to previous methodologies. First, we used little alcohol when diluting odorants. Alcohol is known to stimulate the trigeminal nerve, which could confound any collected data due to activation of an alternative sensory pathway. Therefore, odorants were diluted to 5 % using ethanol and water to adjust their adhesiveness. In our preliminary experiment, we confirmed that all participants were unable to smell the ethanol. Second, the perceived familiarity and pleasantness of the odor was matched for participants. We measured the olfactory detection threshold using isoamyl acetate and allyl caproate, which smell like banana and pineapple, respectively. In our preliminary experiments, we confirmed that all participants were familiar with the two fruits and that they did not dislike the scents, based on Likert scale ratings. Third, our measurement system was kept as simple as possible in order to avoid reflecting attentional differences. Moreover, our system did not require executive function, which has been reported to be impaired in ASD [24, 25].

Thus, we consider that the pulse injection system used in the current study may resolve problems encountered in previous studies. Using this new methodology, the present study was carried out to compare the olfactory detection threshold in children with ASD to age-matched control participants in order to contribute to a better understanding of olfactory abnormalities that are often observed in children with ASD.

## Methods

### Participants

The present study was approved by the ethics committee of the University of Fukui. Participants were TD children and those with ASD; children with ASD were recruited from the University of Fukui Hospital and related clinics. After a complete explanation of the study, all the participants provided written, informed consent. All participants and their guardians agreed to participate in the study. We excluded subjects with an organic smell disturbance, nasal blockage due to sinus/virus compromise, an acute respiratory infection, premenstrual syndrome, or previous history of head injury or illicit substance use. Inclusion criteria of the ASD group were (1) diagnosis of ASD based on the DSM-5 [3], (2) age 8–16 years, and (3) IQ ≧70. Exclusion criteria for the ASD group included medical conditions associated with autism (e.g., FMR1, Rett syndrome, and Shank3). To exclude other psychiatric diagnoses, the Mini-International Neuropsychiatric Interview for Children and Adolescents (MINI Kids) [26] was administered. To obtain data from age-matched TD, healthy schoolchildren aged 8–16 years were recruited as subjects from the community. Control participants had no history or evidence of ASD. To screen control participants for autistic traits, the Childhood Autism Rating Scale-Tokyo Version (CARS-TV) was used. The CARS-TV is the Japanese version of the CARS [27]—one of the most widely used scales to evaluate the degree and profiles of autism in children—and has been determined to have satisfactory reliability and validity [28, 29]. Diagnoses in the ASD group were confirmed by the first author of this report using diagnostic instruments and screening questionnaires including the Pervasive Developmental Disorder—Autism Society Japan Rating Scale (PARS) [30]. The PARS is a diagnostic interview scale for ASD that was developed in Japan, and sub and total scores of this scale correlate with the domain and total scores of the Autism Diagnostic Interview-Revised (ADI-R) [31, 32]. The primary assessment for ASD included interviews regarding the developmental history and symptoms of the children in this group, as well as behavioral observation. Clinical psychologists collected information from parents regarding developmental milestones (i.e., joint attention, social interaction, pretend play, and repetitive behaviors, with onset prior to age 3) and episodes (e.g., behavior throughout school). Information from detailed observations of their interactions with people (particularly non-family members), as well as repetitive behavior (i.e., obsessive-compulsive traits and stereotyped behavior), was provided by other professionals (i.e., teachers and social workers). Since an intellectual level equivalent to that of a 5-year-old is needed to complete the measurements, intelligence testing in children with ASD was performed using the Wechsler Intelligence Scale for Children—Fourth Edition (WISC-IV) [33]. WISC-IV testing was not performed for children with TD because of time constraints. However, all participants were attending mainstream schools with no evidence of intellectual impairment.

### Olfactory measurement

We measured olfactory detection thresholds using a pulse ejection system called the “Fragrance Jet for Medical Checkup” (Fig. 2) (Keio University) [20]. The stimuli used were isoamyl acetate and allyl caproate, which were simple chemicals and, unlike many natural fragrances, were not affected by the preservation method and had the added advantage of being easy to reproduce.

**Fig. 3:**
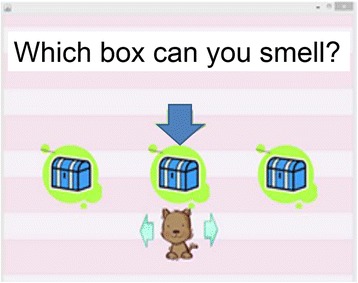
Touch panel display. The touch panel is comprised of three boxes. Three stimuli are presented at random, of which one is scented and the other two are odorless. When participants push a box, odors are given off

The display emitted scents from multiple holes at the same time, allowing the number of simultaneous ejections (NSE) to be set in the range of 0 to 255. Before the experiment, we confirmed that all participants understood the rules of the experiment. We began with an NSE of 80 and an ET of 200 ms based on the results of preliminary experiments and our earlier study [20]. The touch panel system displayed three boxes, each of which contained a stimulus (Fig. 3), and the olfactory detection threshold was assessed using a triple forced-choice procedure where three stimuli were presented at random (one stimulus was scented while the other two were odorless). All children were fascinated by our touch panel display. The presentation was as simple as possible so that all children, including those with ASD, could concentrate during the experiment. The subject’s task was to identify the box that contained the scented stimulus. When the participants pushed one of the three boxes, an odor was given off 3.0 s later. The participant was allowed to push each box up to two times. After two successful trials, the EQ fell by 50 %. This was continued until the participant made a mistake and selected the box that contained an odorless stimulus or until the participant cleared an NSE of 10. We administered the odors from strongest to weakest to maintain the motivation and concentration of all children. The detection threshold was generated after the procedure was completed. Measurements were finished approximately 5 min after the start of the procedure and were influenced very little by lingering scent trails. The olfactory detection threshold was defined using a logarithmic function as the NSE of the last trial. The mathematical process used to determine the olfactory detection threshold is as follows:

$$ \mathrm{Olfactory}\ \mathrm{detection}\ \mathrm{threshold} = { \log}_2\left(\mathrm{N}\mathrm{S}\mathrm{E}\ \mathrm{of}\ \mathrm{last}\ \mathrm{trial}\right)/5 $$

If participants were not able to identify an NSE of 80, their score was 5. Participants that were able to identify an NSE of 80, but could not identify an NSE of 40, were given a score of 4. Finally, if a participant was able to identify an NSE of 40, 20, and 10, their score was 3, 2, and 1, respectively. A subject’s scores could range from 5 (unable to detect even the highest concentration) to 1 (able to detect the lowest concentration) for each odor.

Participants neither ate nor drank anything but water for 30 min prior to testing. Because temperature can influence odor, the temperature of the experimental room was maintained at (21–23 °C) during the study [34]. Experiments were conducted under conditions in which the participant and experimenter were diagonally opposite. A board was placed between them to prevent participants from being able to see the procedures and results. Each participant was required to sit in front of the olfactory display and position their chin on the chin rest such that the distance from the olfactory ejection point to the nose was fixed.

### Statistical analysis

Statistical analysis was performed using the Statistical Package for the Social Sciences (SPSS, version 15.0). Descriptive statistics for the sample were used. The differences of age and CARS score between the groups were analyzed using an independent samples *t* test. The difference in gender proportion was analyzed using the *χ*^2^ test. Differences in olfactory detection threshold were analyzed using the Mann-Whitney *U* test. Pearson product-moment correlation coefficients were used to explore the relationships between age/IQ and the olfactory detection threshold in children with ASD.

## Results

### Demographic data

In total, 43 children took part in this study. All participants completed the experiment in about 5 min. The ASD group included 20 participants (14 males), mean age 13.2 ± 2.1. The total CARS score for the ASD group was 34.1 ± 2.4, and their average IQ score was 100.7 ± 12.1. The TD group included 23 participants (14 males), mean age 12.5 ± 2.2. The total CARS score for the TD group was 17.7 ± 1.3, and all participants were non-smokers. There were no significant differences between groups with regard to mean age (*p* = 0.43) and gender proportion (*p* = 0.53); details are presented in Table 1.

**Table 1:** Descriptive characteristics of the ASD and TD groups

	ASD (*n* = 20)	TD (*n* = 23)	Statistics		
	(M, SD)	(M, SD)	*t* or*χ* ^2^	*df*	*p*
Age (M, SD)	13.2 ± 2.1	12.5 ± 2.2	*t* = 1.100	41	0.278
Gender (males:females)	14:6	17:6	*χ* ^2^ = 0.393	1	0.531
CARS (M, SD)	34.1 ± 2.4	17.7 ± 1.3	*t* = 28.955	41	<0.001

*M* mean, *SD* standard deviation

### Olfactory detection threshold

**Fig. 4:**
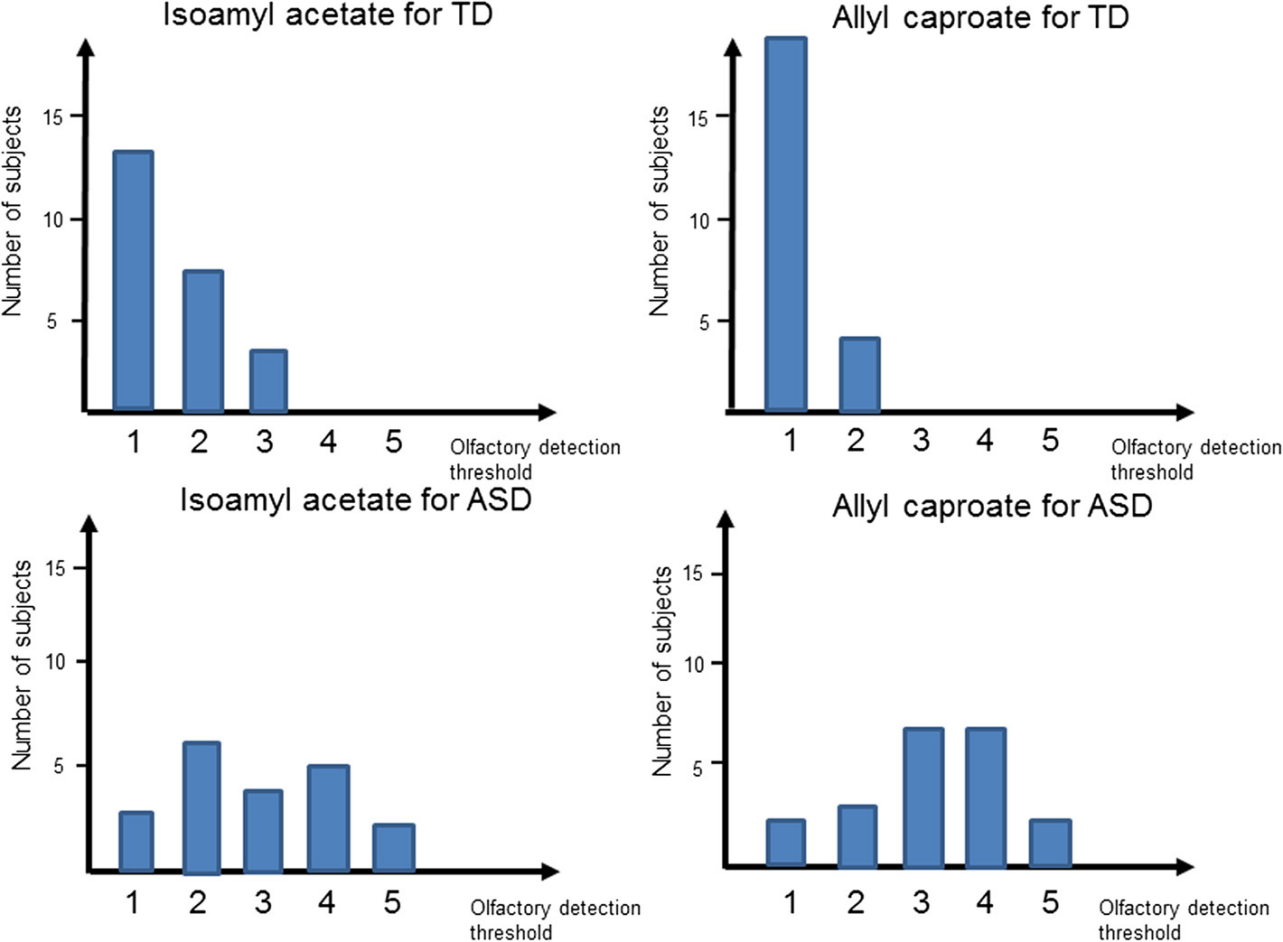
Subjects’ olfactory detection thresholds. Numbers of ASD or TD subjects for each odor stimulus

Olfactory detection thresholds of the ASD group were significantly higher than that of the TD group using both isoamyl acetate (2.85 ± 0.28 vs 1.57 ± 0.15; *p* < 0.001) and allyl caproate ( 3.30 ± 0.23 vs 1.17 ± 0.08; *p* < 0.001) (Table 2). Figure 4 is also a graphic representation of the results. In addition, we did not find any relationship between the olfactory detection threshold of isoamyl acetate and age (*r* = −0.34, *p* = 0.15), IQ (*r* = −0.20, *p* = 0.41) or the olfactory detection threshold of allyl caproate and age (*r* = −0.22, *p* = 0.35), and IQ (*r* = −0.08, *p* = 0.74) in children with ASD.

**Table 2:** Olfactory detection threshold of the ASD and TD groups

	ASD (*n* = 20)	TD (*n* = 23)	Mann-Whitney *U* test
	(M, SEM)	(M, SEM)	*p*
Isoamyl acetate	2.85 ± 0.28	1.57 ± 0.15	<0.001
Allyl caproate	3.30 ± 0.23	1.17 ± 0.08	<0.001

*M* mean, *SEM* standard error of the mean

## Discussion

The aim of this study was to examine olfactory detection thresholds using our newly developed measurement system, which might resolve problems associated with previous laboratory-based sensory psychophysical studies. Using this system, we found that children with ASD were insensitive to both isoamyl acetate and allyl caproate compared to children with TD.

It has been well-described that children with ASD have olfactory abnormalities. More specifically, anecdotal reports and studies using sensory questionnaires have suggested that these children may either show little or no response to olfactory stimuli (high threshold) or, conversely, may be overwhelmed by stimuli (low threshold) [1, 35–37].

These abnormalities also extend to other sensory modalities in that children with ASD may show over-responsivity to unpleasant auditory input, distractibility by background noise, and/or unawareness or slow responses to familiar auditory input [38, 39]. Visual difficulties also include both hyper- and hypo-responsiveness [40, 41]. One plausible theory might be that children with ASD tend to over-focus their attention on details, which results in the superior processing of complex stimuli, whether auditory or visual. However, despite the plethora of studies that have been conducted on disruptions to sensory processing in children with ASD, it remains unclear whether over-attention is the root cause of these abnormalities.

Compared to disruptions of the auditory and visual system, research of the olfactory abnormalities experienced by children with ASD is still in its infancy. In fact, a rough profile of the irregularities to this system has not even been described, let alone a theory proposed for the nature of the abnormalities. Since atypical responsiveness to olfactory stimuli has been reported as the strongest predictor of social impairment in children with ASD [7, 8], understanding the nature of the olfactory abnormalities is crucial for the optimal intervention of these children. These factors prompted us to conduct the present study using our newly developed jet pulse injection system for determining olfactory thresholds. Our rational was that, in order to understand the olfactory abnormalities experienced by children with ASD, we first needed to measure a simple detection threshold. In the present study, by using newly developed methodology, we were able to show that children with ASD exhibited high thresholds for the two stimuli studied. As previously described, earlier studies in this field have reported inconsistent results, but methodological differences hamper direct comparison [13–16]. Suzuki et al. [15] used 1-butanol for olfactory testing, Dudova et al. [14] and Tavassoli and Baron-Cohe [16] used Sniffin’ Sticks [18] with n-butanol, and Ashwin et al. [13] employed the alcohol sniff test [19] with isopropyl alcohol. Although these previous studies used alcohol as the odorant, each used different kinds of alcoholic odorants in olfactory detection threshold testing, which could lead to inconsistent results [42]. In addition, one study [14] tested children, while three others examined adult subjects [13, 15, 16]. Chronologic aging plays an important role in olfactory research in ASD [43]. Since it has been suggested that the olfactory system develops differently in ASD compared to controls [44, 45], age differences may explain the discrepant results. It should be noted that some of those studies were focused on the identification of odors, not on the detection of olfactory thresholds. A recent study conducted by Ashwin et al. [13] examined thresholds for olfactory detection using a carefully designed method. They used isopropyl alcohol for odor and also employed methods to prevent trigeminal nerve activation and to familiarize the subjects with the alcohol odor. They also performed an experiment where air movement was minimized; windows remained closed and covered throughout, and there was no air control mechanism. However, their results contradicted the results reported here. One explanation for this discrepancy might be the difference in stimuli used. In fact, Ashwin et al. [13] suggested that the pleasantness level of odors for people with ASD could also affect their detection threshold for that odor.

Sensory abnormalities may be a key physiological factor underlying social impairments that are associated with ASD. It has been proposed that early perceptual capacities of individuals with ASD may set up a cascade of development deficits that contribute to the poor social skills seen at older ages. Given the recent evidence that atypical responsiveness to olfactory stimuli is the strongest predictor of social impairment in children with ASD [7, 8], our results might help to elucidate the nature of ASD-related social impairment. For instance, odor has been shown to have influence on mood as well as autonomic, endocrine, and immune functions [46]. Odors have also been shown to play an important role in inducing emotional reactions, imitating the action of others, and regulating social interactions [47–49]. Thus, insensitivity to some odors might have a tremendous impact on children with ASD, which might explain findings reported by Lane et al. [8] describing that olfactory disturbances could predict communication competence and maladaptive behavior. Since both social-emotional and sensory functions are related to the amygdala, research on olfactory detection may lead to further elucidation of the neurobiology of ASD. In addition, Brewer et al. [50] and Hrdlicka et al. [51] have suggested that examination of olfactory disturbances could provide early markers of ASD. Thus, assessment of olfactory detection thresholds might lead to the development of useful diagnostic tools, increasing service and therapeutic efficacy for children with ASD. Moreover, investigating olfactory detection thresholds in children with ASD might contribute to our understanding of the neurobiology of ASD and related disorders. Future studies using a variety of olfactory stimuli, participants of different ages, and autistic traits and relationships will be fruitful.

A number of study limitations must be acknowledged. First, we did not perform exact IQ testing in the control group, and relied only on normal school performance of the subjects that enrolled in this experiment. However, all controls were attending mainstream schools with no evidence of intellectual impairment. We confirmed that 5-year-old children with average intellectual and verbal competency could complete the measurements in our preliminary experiments. The present results also demonstrate that IQ is not correlated with the olfactory detection threshold for children with ASD. One previous study suggested that cognitive factors are unrelated to performance on olfactory detection threshold tests [9]. However, since our methodology relies on verbal comprehension and communication, the subjects’ verbal abilities could affect the results. In relation to this point, two recently published studies employed unique approaches that do not rely on verbal communication [52, 53]. Aguillon-Hernandez et al. [52] used an objective, vision-based approach to evaluate odor identification and reported that visual exploratory behavior may be influenced by olfactory identification. Rozenkrantz et al. [53] used a computer-controlled air-dilution olfactometer equipped with a custom-designed double-barreled pediatric nasal cannula and found that the sniff response was linked to social impairment in ASD. Because the methodologies used in these studies do not rely on verbal communication, they can be used in non-verbal children with more severe forms of ASD, which greatly expands the types of subjects who can participate in research. In future studies, pairing our odor presentation method with these innovative methodologies could yield results that are more robust. In addition, it should be emphasized that Aguillon-Hernandez and colleagues focused on odor identification, the evaluation of which was dependent on verbal labeling and semantic memory in conventional methodologies [54, 55]. An important future line of research would be to study both odor identification and threshold detection in the same group of subjects, since an important dissociation could occur between identification and sensitivity across the spectrum. Second, our study employed a relatively small number of participants (*n* = 43), which included 20 children with TD and 23 children with ASD. Moreover, most of our sample consisted of males. Given the sex differences in olfactory functioning that have been identified in healthy subjects [56], future research should examine larger samples and include more female participants. Third, we used only two olfactory stimuli in the present work. Moreover, both stimuli were associated with fruit smells, which limits making generalizations about other odorants. Therefore, validation of the present findings using a more sophisticated method in a larger independent cohort is needed.

## Conclusions

In summary, this study showed that the olfactory detection threshold for children with ASD was higher than that for children with TD. We were able to determine this using a pulse ejection system, which could measure detection thresholds more precisely than previous laboratory-based sensory psychological studies. Future studies of olfactory processing in individuals with ASD using this methodology may reveal important links between brain function, clinically relevant behavior, and treatment.

## Abbreviations

ASD: autism spectrum disorder
CARS: Childhood Autism Rating Scale
DSM-5: Diagnostic and Statistical Manual of Mental Disorders, 5th Edition
EQ: ejection quantity
EQUT: ejection quantity per unit time
ET: ejection time
NSE: number of simultaneous ejections
TD: typical development
